# SARS-CoV-2 Detection via RT-PCR in Matched Saliva and Nasopharyngeal Samples Reveals High Concordance in Different Commercial Assays

**DOI:** 10.3390/diagnostics13020329

**Published:** 2023-01-16

**Authors:** Karoline Almeida Felix de Sousa, Carolina Kymie Vasques Nonaka, Renata Naves de Ávila Mendonça, Verena Neiva Mascarenhas, Thamires Gomes Lopes Weber, Carlos Gustavo Regis Silva, Ana Verena Almeida Mendes, Ricardo Khouri, Bruno Solano Freitas Souza, Clarissa Araújo Gurgel Rocha

**Affiliations:** 1 Gonçalo Moniz Institute, Oswaldo Cruz Foundation (FIOCRUZ), Salvador 40296-710, Brazil; 2 Center for Biotechnology and Cell Therapy, D’Or Institute for Research and Education (IDOR), Salvador 41253-190, Brazil; 3 São Rafael Hospital, Salvador 41253-190, Brazil; 4 Faculty of Medicine, Federal University of Bahia, Salvador 40110-100, Brazil; 5 Faculty of Dentistry, Federal University of Bahia, Salvador 40110-150, Brazil

**Keywords:** COVID-19, saliva, diagnostic method, SARS-CoV-2, RT-PCR

## Abstract

Background: Self-collected saliva samples can increase the diagnostic efficiency and benefit healthcare workers, patient care, and infection control. This study evaluated the performance of self-collected saliva samples compared to nasopharyngeal swabs using three commercial kits for the qualitative detection of severe acute respiratory syndrome coronavirus 2 (SARS-CoV-2). Methods: Matched nasopharyngeal and saliva samples were collected from 103 patients with either asymptomatic or symptomatic COVID-19. Both samples were evaluated using three commercial kits (TaqCheck, Allplex, and TaqPath). To evaluate sample stability, viral RNA extraction was performed in the presence or absence of an RNA-stabilizing solution. Storage conditions, including the duration, temperature, and stability after freezing and thawing of the samples, were also evaluated. Results: All the saliva samples showed 100% concordance with the nasopharyngeal swab results using TaqCheck and Allplex kits, and 93% using TaqPath kit. No difference was observed in the samples that used the RNA-stabilizing solution compared to the group without the solution. The *Ct* values of the freeze–thawed samples after 30 days were higher than those on day 0; however, the results were consistent the fresh samples. Conclusion: The high concordance of SARS-CoV-2 detection via reverse transcription–polymerase chain reaction (RT-PCR) in matched saliva and nasopharyngeal samples using different commercial assays reinforces the concept that self-collected saliva samples are non-invasive, rapid, and reliable for diagnosing SARS-CoV-2 infection.

## 1. Introduction

Implementing efficient mass screening strategies with molecular tests is crucial to ensure the extensive detection of severe acute respiratory syndrome coronavirus 2 (SARS-CoV-2), monitor viral dissemination, and control further outbreaks [1,2]. Reliable, sensitive, and affordable tests for the detection of SARS-CoV-2 are essential for public health responses to the COVID-19 pandemic. The real-time reverse transcriptase–polymerase chain reaction (RT-PCR)-based detection of SARS-CoV-2 is the “gold standard” for the diagnosis of COVID-19. Several RT-PCR kits have been developed based on the conserved regions of the SARS-CoV-2 genome [3,4,5]. Owing to their high sensitivity and specificity, both upper and lower respiratory tract samples have been considered as standard sampling materials. However, collecting samples from the respiratory tract requires dedicated infrastructure, trained personnel, and well-established procedures to avoid contamination and risk of the infection of healthcare workers, which are not always accessible, especially in developing countries. Moreover, adherence to testing protocols requiring repeated nasopharyngeal (NP) swab collection may be low because this procedure may be associated with different levels of discomfort, cough, bleeding, rhinitis, sneezing, and/or vomiting [6,7,8,9].

Saliva has emerged as a potential alternative to NP swab samples with comparable levels of sensitivity and specificity [10]. Saliva sampling offers several advantages including fewer material resources and infrastructure [11,12]. In addition, self-collected saliva is a simple, non-invasive method that is well-tolerated by children, the elderly, and people with disabilities [13]. In 2020, the RT-PCR assay for SARS-CoV-2 virus detection in saliva was approved by the US Food and Drug Administration (FDA) [14], and a recent study showed high positive agreement (94%) between saliva by SalivaDirect and NP swabs using a commercial RT-PCR kit [15]. Accordingly, a meta-analysis study demonstrated a concordance rate of more than 90% for saliva and NP swabs, with high sensitivity and specificity [10].

Currently, the American Centers for Disease Control and Prevention and the European Centre for Disease Prevention and Control authorize the use of oral swabs or saliva as specimens for SARS-CoV-2 diagnosis. Saliva testing is already a well-accepted protocol for SARS-CoV-2 detection in several countries, including the Republic of Korea, Germany, and Japan [16].

Despite the amount of evidence suggesting the comparable performance of the NP swabs and saliva samples, there is still a need to characterize whether patient profile, severity of symptoms, and time between disease onset and sample collection may affect the efficacy of these tests. In addition, practical questions regarding kit performance and pre-analytical factors that could affect sample stability have not been sufficiently explored. Here, we compared the diagnostic performance of three RT-PCR-based commercial kits for the qualitative detection of SARS-CoV-2 in salivary samples with matched NP swab samples and correlated the patients’ clinical data.

## 2. Materials and Methods

### 2.1. Patients and Sample Collection

This study was approved by the Institutional Review Board (Number CAAE:38580920.6.3001.0040). All the included patients were tested according to their clinical indications. A total of 103 symptomatic and asymptomatic patients were included in this convenience cohort study, who were admitted to the emergency room at São Rafael Hospital (Salvador, Bahia, Brazil) from 16 March to 6 May 2021. Informed consent was obtained from all the patients. Demographic data, comorbidities, and symptoms were collected from the electronic medical records.

Paired saliva and NP swab samples were collected from these patients. As a service offered by the São Rafael diagnostic laboratory, the NP swab samples were collected by a qualified technician and stored at −20 °C until they were processed further. Approximately 5 mL of saliva was collected by the patients themselves in a certified DNase and RNase conical polypropylene sterile graduated tube (50 mL). The participants were asked to avoid eating and drinking 30 min before sampling. After collection, the saliva samples were stored at −80 °C until they were processed further.

### 2.2. Sample Processing and RT-PCR for SARS-CoV-2

The saliva samples were processed following the recommendations of a reference kit for SARS-CoV-2 detection in saliva samples, the TaqCheckSARS-CoV-2 Fast PCR assay kit (Thermo Fisher Scientific, Pleasanton, CA, USA). Saliva samples were incubated with Tween-20 Detergent (1%)/TBE Buffer (2×) (Thermo Fisher Scientific, Carlsbad, CA, USA). An amount of 100 μL of saliva was incubated at 95 °C for 30 min, following which, the sample was vortexed until it appeared homogeneous; then, it was mixed with 100 µL of TBE/Tween mix (20 µL of TBE Buffer (10×) + 10 µL of Tween 20 Detergent (10%) + 70 µL of nuclease-free water).

For stability assessment, 28 positive samples were randomly selected, and in a final volume of 1 mL, 1:2 DNA/RNA Shield (Zymo Research, Irvine, CA, USA) solution was added and compared with 1 mL of the paired sample without RNA Shield. Both samples were stored at −80 °C for 30 days. Viral RNA was extracted after 30 days using the MagMAX Viral/Pathogen II (MVP II) Nucleic Acid Isolation Kit (Thermo Fisher Scientific, Austin, TX, EUA), according to the manufacturer’s recommendations. The automated KingFisher sample purification system (Thermo Fisher Scientific, Austin, TX, EUA) was used. Similarly, 30 positive samples were randomly selected and stored without RNA Shield at −80 °C for 30 days, followed by processing with 2× TBE/1% Tween-20 mix.

**Figure 1:**
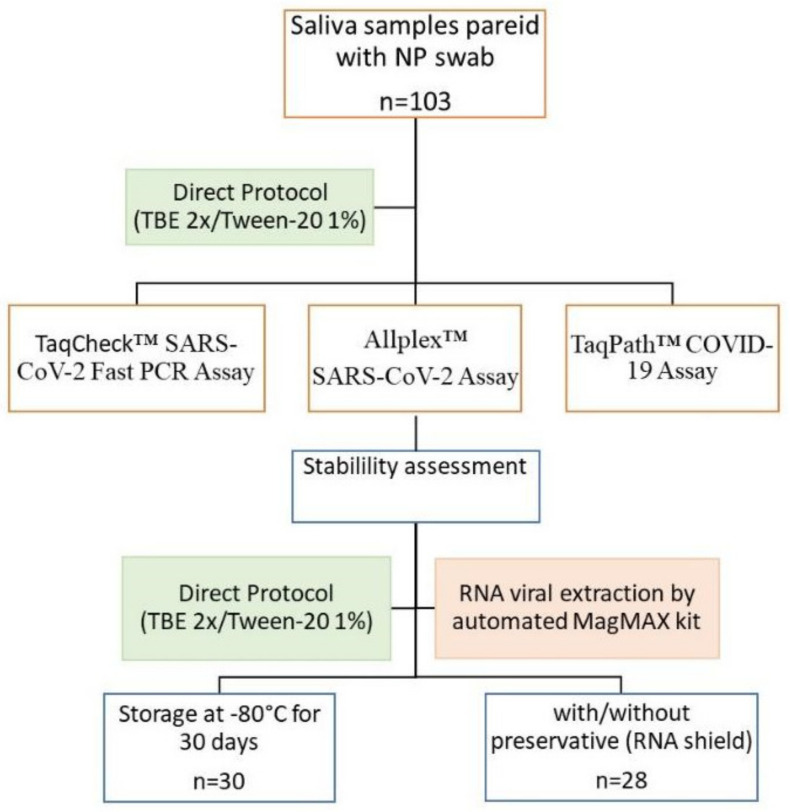
Flowchart of the study design.

The performances of three commercial SARS-CoV-2 detection kits, Allplex SARS-CoV-2 Assay kit (Seegene, Songpa-Gu, Seoul, Republic of Korea), TaqPath COVID-19 CE-IVD RT-PCR kit (ThermoFisher Scientific, Pleasanton, CA, USA) and TaqCheck SARS-CoV-2 Fast PCR assay kit (Thermo Fisher Scientific, Pleasanton, CA, USA), were evaluated and compared according to the study design described in Figure 1. The standard RT-PCR assay for SARS-CoV-2 virus detection was performed using Applied Biosystems™ 7500 Fast (Thermo Fisher Scientific, MA, USA), and software v2.3 was used for the analysis of the results. N-gene amplification via RT-PCR was used to compare the *Ct* values of the SARS-CoV-2 detection kits. The cycle threshold values were set at *Ct* < 40 as an indicator of positive test results.

### 2.3. Statistical Analysis

The results obtained from saliva samples were compared with those obtained from NP swab samples. The kappa coefficient was used to assess the agreement between the results. All the statistical tests were performed using the GraphPad Prism version 9.0 software (GraphPad, La Jolla, CA, USA).

## 3. Results

In this study, 103 paired samples (NP swab and saliva) were obtained from patients admitted to the emergency room of a private hospital (São Rafael Hospital, Salvador, Bahia, Brazil) with flu-like symptoms (n = 78) or asymptomatic subjects who reported coming in close contact with COVID-19 patients in the past few days (n = 25). Symptomatic patients underwent sample collection at a median of 5 days from the onset of illness, and among them, 56.4% (n = 44) tested positive for SARS-CoV-2. Cough (n = 30, 65.2%), headache (n = 24, 52.2%), and fever (n = 21, 45.7%) were the most frequent symptoms reported. The other clinical symptoms and demographic data are shown in Table 1.

### 3.1. Comparative Performance of Self-Collected Saliva Samples via Extraction-Free Protocols

After saliva collection, we evaluated an extraction-free protocol using a solution of Tween-20 Detergent (1%)/TBE Buffer, followed by detection using two commercially available kits for the molecular diagnosis of SARS-CoV-2 via RT-PCR, Allplex and TaqPath. Our aim was to evaluate the cost reduction and optimization of the diagnostic test by eliminating the viral RNA extraction step. The samples were also processed with a commercial kit, TaqCheck, established for saliva samples, and the results were compared with NP samples. Allplex and TaqPath have been validated for NP samples and are routinely used in SARS-CoV-2 diagnostics by the clinical laboratory of São Rafael Hospital. However, this commercial kit had not been validated for SARS-CoV-2 detection in saliva samples by manufacturers at the time of the initiation of the present study. When comparing Allplex and TaqPath with TaqCheck in saliva samples, the kappa coefficients were observed to be 1.0 and 0.9, which corresponded to excellent agreement (n = 46 positive, n = 57 negative) (Table 2).

**Figure 2:**
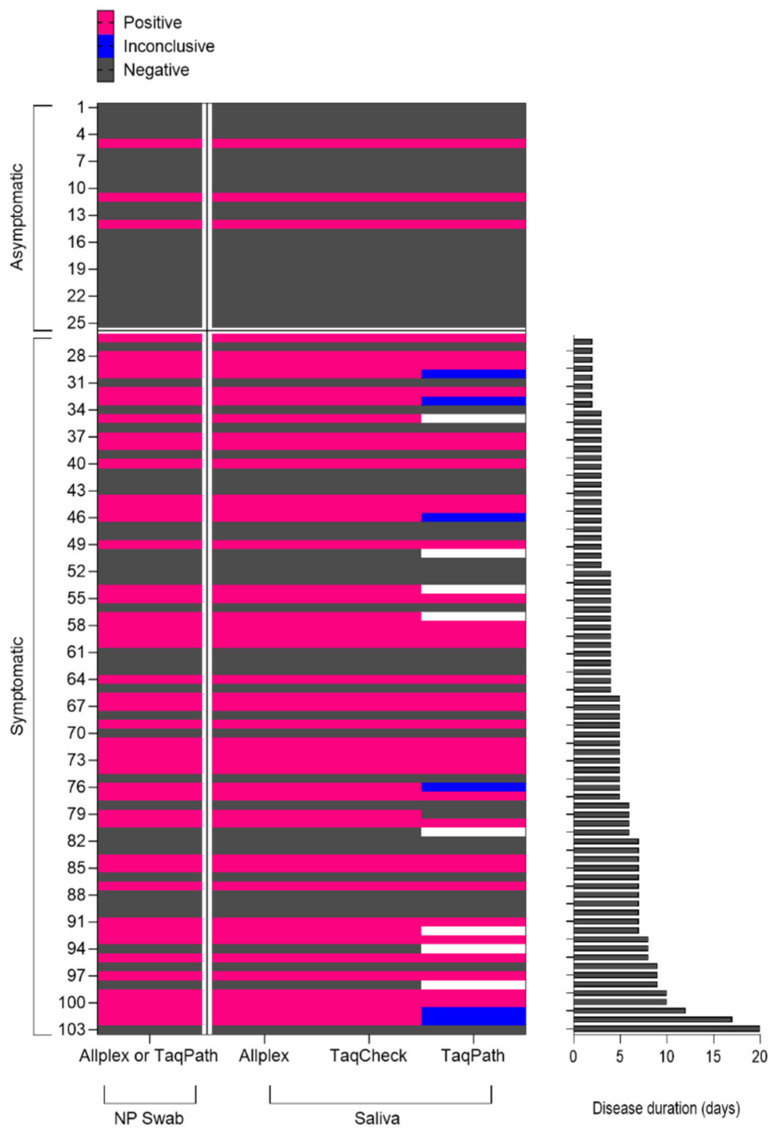
Heat map showing the RT-PCR results for detecting SARS-CoV-2 in paired NP swab and saliva samples, obtained from asymptomatic patients (contactors or not) and symptomatic patients with collections performed after different days of symptoms. Positive tests (pink), negatives (black), inconclusive (blue), and not tested (white). Abbreviations: RT-PCR: reverse transcription–polymerase chain reaction; NP: nasopharyngeal.

We observed the same kappa coefficients (n = 46 positive and n = 57 negative) when comparing the performance of Allplex and TaqPath in saliva samples with the NP swab. We also evaluated the possible influence of the time between sample collection and symptom onset on test sensitivity using *Ct* values (Figure 2). NP samples presented lower median *Ct* values than saliva samples, reaching statistical significance for samples collected 10 days after symptom onset.

### 3.2. Evaluation of the Stability of Stored Saliva Samples

Next, we evaluated the stability of 30 randomly selected samples with positive results stored at −80 °C for 30 days without RNA Shield stabilizing solution. The results were 100% in agreement after 30 days since we detected SARS-CoV-2 with *Ct* < 40 in all the samples with Allplex, with median *Ct* = 27.8 in stored samples and *Ct* = 25.0 in fresh samples.

The influence of the RNA stabilization buffer was evaluated using RNA Shield with saliva and storage at −20 °C for up to 30 days compared to paired samples without RNA Shield and without the purification step. A viral RNA purification step was performed only when the RNA Shield was added to the samples, using the Allplex SARS-CoV-2 detection kit. However, we observed a benefit when an RNA-stabilizing solution was added. Lower *Ct* values were observed with RNA Shield and RNA purification compared to those without RNA shield and an extraction-free protocol, with median *Ct* = 18.4 and *Ct* = 27.2, respectively.

## 4. Discussion

In this study, we observed 100% concordance between the RT-PCR of saliva samples for SARS-CoV-2 detection and NP swabs using the Allplex SARS-CoV-2 detection kit and the TaqCheckSARS-CoV-2 Fast PCR kit. Our results are in accordance with those of other studies that support saliva as an alternative sample for COVID-19 diagnosis [17]. One study has shown that it was possible to isolate and cultivate SARS-CoV-2 from saliva samples [18]. In addition to the equivalent sensitivity, the lower cost, the simplicity of self-collection, and increased patient comfort make saliva fluid sampling more advantageous than NP swabs. Furthermore, we demonstrated that RNA extraction-free protocols can be used with the Allplex SARS-CoV-2 detection kit in the absence of preservatives for the collection of the saliva samples.

Most people infected with SARS-CoV-2 develop mild-to-moderate respiratory illnesses. The risk of severe illness increases in older adults and those with certain underlying medical conditions [19]. In our study, too, the prevalence of fever, cough, and headache was frequently observed in both the positive (COVID-19 detected) and negative (COVID-19 not detected) evaluated groups. All the individuals included in this cohort study were either mild or subclinical. Interestingly, our study demonstrated the presence of viral RNA in asymptomatic patients. SARS-CoV-2 testing, such as contact tracing, is a strategy to identify people infected with SARS-CoV-2 [20]. Consistent with our findings, it was demonstrated earlier that peak positivity in asymptomatic RT-PCR occurs between 1 and 3 days after infection [21]. Saliva is a reliable source for diagnosing COVID-19 by detecting viral RNA in symptomatic and asymptomatic individuals, although it shows less sensitivity than NP swabs.

Although RT-PCR is considered the gold-standard method for SARS-CoV-2 detection, it is important to note that this method may not always provide a 100% confirmation and thus may have both better and worse performance among COVID-19 diagnostic assays. In the early stages of the pandemic, a study evaluated some primer–probe sets in SARS-CoV-2 RT-PCR diagnostic assays and demonstrated a difference in sensitivity between them [22]. Another interfering condition already described is that the probability of detecting SARS-CoV-2 varies throughout the infection [23]. In agreement with the literature, we observed similar results in NP swab and saliva specimens using the Allplex SARS-CoV-2 assay or the TaqCheckSARS-CoV-2 Fast PCR. The detection of SARS-CoV-2 in saliva samples has been shown to help monitor viral load dynamics over time, indicating that the symptom of the highest viral load occurs during the beginning of the first week and decreases over time [24]. Despite using different prime–probe sets, all the protocols presented results that were in agreement with the literature, with a lower *Ct* value at the onset of symptoms with increasing *Ct* values over time for both samples. Additionally, we observed a benefit in the *Ct* value when adding the RNA-stabilizing solution together with the viral RNA purification step, especially during the storage of the samples.

Finally, this study had some limitations. First, individuals with severe symptoms and pediatric patients were not included. Second, the NP swabs were processed within 12 h as part of the São Rafael Hospital laboratory’s routine protocol, and in eight samples, the number of NP swabs and saliva samples was not sufficient for running all the tests in parallel (Figure 2). Saliva samples were frozen for at least one week, which may have interfered with the sensitivity of the results. Third, we did not include serological confirmation of COVID-19 in all the cases.

## 5. Conclusions

In conclusion, saliva is a sensitive and viable sample source for diagnosing COVID-19. Our study shows that it is possible to identify asymptomatic infectious individuals without the need for RNA extraction and an RNA-stabilizing solution, thus making diagnosis with self-collected saliva samples a promising, noninvasive, and well-tolerated method in routine laboratory tests. Finally, we reinforced three factors that can impact diagnostic performance: (I) the quality of the collection sample, (II) collection time in relation to the viral load, and (III) cross-contamination.

## Figures and Tables

**Table 1:** Clinical and demographic characteristics of the patients (n = 103) and results of the detection of SARS-CoV-2 in nasopharyngeal swab via RT-PCR.

	Detection of SARS-CoV-2	
	Positive (n = 46)	Negative (n = 57)
Age (median) years	40 (18–79)	42 (18–79)
Male n(%)	29 (63.04)	24 (42.10)
Symptomatic n(%)	44 (99.65)	34 (59.65)
Fever n(%)	21 (45.65)	11 (19.29)
Cough n(%)	30 (65.21)	16 (28.07)
Sore throat n(%)	10 (21.73)	10 (17.54)
Dyspnea n(%)	15 (32.60)	6 (10.52)
Headache n(%)	24 (52.17)	19 (33.33)
Myalgia n(%)	20 (43.47)	13 (22.80)
Anosmia n(%)	7 (15.21)	1 (1.75)
Diarrhea n(%)	2 (4.34)	10 (17.54)
Vomiting n(%)	3 (6.52)	4 (7.01)

RT-PCR: reverse transcription–polymerase chain reaction.

**Table 2:** Comparison between three commercial kits for detection of SARS-CoV-2 in saliva samples (n = 103) by RT-PCR using Cohen’s kappa coefficient.

SARS-CoV-2 Detection Kit	Kappa	95% CI
TaqCheck (saliva) vs. Allplex (NP)	1.0	1.0–1.0
TaqCheck (saliva) vs. Allplex (saliva)	1.0	1.0–1.0
TaqCheck (saliva) vs. TaqPath (saliva)	0.923	0.849–0.997
Allplex (NP) vs. Allplex (saliva)	1.0	1.0–1.0
Allplex (NP) vs. TaqPath (saliva)	0.923	0.849–0.997

RT-PCR: reverse transcription–polymerase chain reaction; NP: nasopharyngeal.

## Data Availability

Not applicable.
